# American Protological Society

**Published:** 1910-08

**Authors:** 


### Abstracts and Selections.

### AMERICAN PROTOLOGICAL SOCIETY.

The following is an abstract of papers read at the twelfth annual meeting of the American Proctological Society, St. Louis, June, 1910:

### Malformation of the Anus and Rectum; Report of Four Cases.

BY ALOIS B. GRAHAM, A. M., M. D., INDIANAPOLIS, IND.

Congenital malformations demand prompt surgical treatment. Many cases are never reported and the percentage is evidently much larger than statistics indicate. These malformations are sufficiently uncommon and interesting to warrant placing every case on record. Report of four cases.

Case 1.—White male child, born with no trace of an anus, and in whom careful dissection and exploration failed to find any trace of a rectum. Colostomy was suggested but the parents refused their consent. Child died four days later. Autopsy refused.

Case 2.—Colored male child, age 5 years, born with a complete obstruction of the anus by a membranous diaphragm, which was perforated by the attending physician. Examination revealed a dense stricture, almost impermeable, involving the entire anal canal. The interesting point was the presence of a hypospadias through which feces had escaped for two years. The communication between the rectum and urethra was the result of ulcerations above the stricture rather than defective embryological development. Surgical treatment was refused.

Case 3.—Colored female child, age fifty-six days, in whom examination revealed a well-formed anus and a protruding or bulging imperforate rectum. A photograph shows a pronounced distension of the abdomen, the result of fifty-six days intestinal obstruction. Posteriorly, the rectum had no attachments, and the finger could be introduced easily behind the bulging imperforate gut, through the anal canal, into a blind pouch. A fistulous opening was found in the vagina just behind the hymen. The meconium and a small quantity of feces had escaped through this opening. The protruding rectal mucosa was dissected from its attachments and excised. The rectal mucosa was then sutured to the free skin at the anal margin, except for one-eighth of an inch posteriorly. This was used for drainage in case the blind pouch became infected. This patient made a good recovery. At the last examination, which was three months following operation, the finger could be introduced easily into the rectum, the stools were normal, and sphincteric control was good. The fistulous opening into the vagina was closed, and the posterior rectal mucosa was firmly united to the skin at the anal margin. With the exception of an abdomen, which seemed to be a trifle prominent for one of its age, the child appeared normal.

Case 4.—White child, one of twins, age forty-two hours, in whom examination revealed an imperforate urethra and no trace of an anus. Penis and scrotum were well developed, but neither testicle could be palpated. Careful dissection and exploration failed to find any trace of a rectum. A two-inch incision was made in the median line just above the pubic, but no bladder could be found. Decided to perform a colostomy or sigmoidostomy. A portion of what was supposed to be the sigmoid was opened and a large quantity of meconium escaped. Exploration revealed a pouch which appeared of much larger dimensions than a normal colon or sigmoid should be. Operation was completed, and yet our inability to find the bladder made the case a hopeless one. Child died twenty-four hours later. At autopsy no bladder was found. The entire large intestine was removed. This case is of interest from the point of view of defective development. The pouch-like termination of the intestine might well be termed a monstrosity. The writer is inclined to believe that it is one of those rare cases in which the colon or sigmoid opens into the uterus. While the local examination revealed a male child, with the exception of being able to palpate the testicles, the examination of the specimen removed at autopsy revealed marked evidence of the female generative organs. The child was a transvere hermaphrodite—namely, one in whom the external genitals seem to be of one sex and the internal of the other. Report of examination of specimen stated that the pouch-like termination of the intestine is formed of three organs, namely, the bladder, uterus and rectum. (Specimen shown.)

### The Use of Quinine and Urea Hydrochloride as a Local Anesthetic in Ano-Rectal Surgery.

BY LOUIS J. HIRSCHMAN, M. D., DETROIT, MICH.

Dr. Hirschman presented to the society a report of his work with quinine and urea hydrochloride as a local anesthetic in ano-rectal surgery. The cases operated upon were as follows:

Acute thrombotic hemorrhoids, 10; internal hemorrhoids, 22; interno-external hemorrhoids, 7; external hemorrhoids, 10; fistula-in-ano, 14; abscess peri-anal, 7; fissura-in-ano, 7; excision of scar tissue, 3; Ball's operation (pruritus ani), 2; hypertrophied papillae, 16; inflamed Morganian crypts, 4. Total, 102.

He reported perfect results as far as operative anesthesia was concerned in every case, and in but seven cases was there any post-operative pain. He uses the 1 per cent solution of quinine and urea hydrochloride in all of his cases of ano-rectal surgery, where suturing of the skin is not required.

The technic of administration as employed by Hirschman is the same as that used with weak solutions of cocaine and eucaine. He describes this technic in detail. He believes that the substitution of quinine and urea hydrochloride for any of the other anesthetic salts hitherto employed will be found eminently satisfactory in all cases of ano-rectal surgery, where suturing of the integument is not required. He sums up its advantages over the other anesthetic drugs as follows:

1. It is soluble in water.
2. It can be sterilized.
3. It is equal to cocaine in anesthetic power.
4. It is absolutely non-toxic.
5. It has a pronounced hemostatic action.
6. Post-operative anesthesia lasts from four hours to several days.
7. It is inexpensive and most always available.

---

### Atony of the Rectum.

---

BY WILLIAM M. BEACH, M. D., PITTSBURG, PA.

---

Dr. Beach stated that atony or sluggishness of the rectum signifies the inability to expel its contents by reason of impaired musculature, ligimentation or innervation, and further that the musculature in the rectum proper, or that portion above the plane of the levator ani is entirely involuntary whose inertia must therefore be due to some inherent factor.

On the contrary, the anal canal, which is made up for the most part of the voluntary fiber, has most to do with the expulsive act, the normal function of which depends chiefly upon the muscular automaton that is intact, proper innervation and psychic influence.

The physiologic rectum depends upon (1) an unobstructed canal, (2) firm ligaments, and (3) a well-developed rectal sense residing in the anal canal. Factors contributing to atony are (a) traumatism to the perineal body, (b) disease in the anal canal, (c) enteroptosis secondary to general systemic conditions or local anatomic anomalies, (b) the abuse of injections and drastic catharsis, (e) disease in adjacent organs, as prolapsed uterus, adhesions, neoplasms, appendicitis, prostatitis, circulatory disturbance as engorged portal vessels and primary gastric diseases. (f) atony may be the sequel to leusis or senility. The treatment is that of constipation being guided by the cause. Alterative, dietetic and mechanical agencies are to be invoked.

---

### Villous Tumor of the Rectum.

BY T. CHITTENDEN HILL, M. D., BOSTON, MASS.

The author stated that a villous tumor of the rectum is very uncommon and but few cases have been recorded in current literature. B. Merrill Ricketts reported a case before this society in 1907 and states that but "sixty-two cases have been reported, nine of which have been by six American authors." Since then I have been able to find but one case reported by Vautrin—(L'Review de la Gynecologia). His article is the most accurate and painstaking observation to be found on the subject.

It is rather difficult to arrive at any conclusion as to their relative frequency by studying the reported cases or by searching hospital reports, as these border-line tumors are generally very loosely classified. Probably the most accurate data at our disposal may be had from St. Mark's Rectal Hospital, London, in which twenty-five villous tumors are tabulated among 42,343 patients with rectal ailments.

The chief point of interest about these tumors is that a certain percentage of them show a marked tendency to undergo malignant degeneration. From the histories of the thirteen cases cited by Ricketts, including one of his own, we learn that three recurred and three did not. Those with a broad base, later became malignant, while those with a pedicle did not. Of the other seven cases no mention was made as to the final outcome.

Goodsall and Miles have had twelve cases—eight in men and four in women, of which number two ultimately became carcinomatous.

From careful study of these cases and several others the author believes that if there is a distinct pedicle without infiltration of the adjacent mucous membrane, tumors of this type are generally benign and if completely removed by ligation, or otherwise, there is but little likelihood of their recurring. On the other hand, if the base is broad, whether there be induration or not, a total extirpation of the rectum should be advised.

Another point of some interest borne out by a study of these cases is that the longer the condition has existed the less likely is it that the growth will prove malignant. The case now reported seems to bear out this statement.

Mrs. M., 40 years of age, was referred by Dr. J. H. Vaugn, of Everett, Mass., January 5, 1907. She was well-nourished, weight about normal, but anemic, with sallow complexion. Had had indigestion for years but in other respects was in good health. For the past six years had noticed small rectal hemorrhages. During the year pervious the hemorrhages had become more profuse and the mass was always protruded at the anus during defecation and even after slight exertion when walking.

She had to go to the toilet several times during the day and to get up two or three times at night, when she would pass one-half cupful of blood-stained mucus; also considerable mucus would at times escape with flatus. For two months, tenesmus had been present nearly all the time. She did not complain of anal or sacral pain.

Rectal examination. Sphincters, peri-anal skin and anal canal were perfectly normal. In the rectum was felt a slippery growth with a band-like pedicle one inch wide by one-half inch thick, attached obliquely with the long axis of the rectum. By careful manipulation the writer was able to bring outside the anal orifice, a lobulated cauliflower like mast, the size and shape of a large English walnut, from which there was a gentle oozing of blood while it was held outside by the sphincters.

Operation January 8, 1907. The sphincters were stretched after infiltration with one-quarter of 1 per cent cocaine solution and the mass drawn down with the finger and the pedicle infiltrated and clamped about half an inch from the margin of the tumor.

The pedicle was then transfixed on the proximal side of the clamp and ligated with pagenstechere No. 5 in three sections and the pedicle cut away on the distal side. An ounce of bloody mucus escaped from the anus during the dilation.

The operation was easily performed and with but little discomfort to the patient under local anesthesia.

Over three years have now elapsed since the case was operated upon and as yet there is no sign of recurrence.

The report of Dr. Louis Hoag upon specimen, January 8, 1907, was as follows: "Pedunculated cauliflower tumor of flattened spheroidal form of pale brownish red color and 4x3½ cm. in size.

"Surface quite regularly broken by deep marrow pits and furrows between and among hundreds of small hemispherical ovoid and spindle-shaped lobules ranging from 1 to 3 mm. in diameter. Such are soft, juicy but not necrotic and of uniform pale brownish red color. Surface always smooth and glistening. Irregularly distributed are deeper clefts outlining pyramidal divisions of the tumor, each bearing upon its base, which is directed outward, a number of the lobules just described.

"Toward the periphery of the cross-section of the tumor the lobules are of uniform-soft consistency and of uniform pale-brown red color. Centrally the pale pedicles, which are about 4 mm. in diameter, enter the tumor at a sort of hilus and its white fibrous tissue bearing numerous small blood-vessels spreads out to be finally lost in the similar tissue of the apices of the various pyramidal divisions of the tumor."

---

### Significance of Rectal Hemorrhage.

---

BY LOUIS J. KROUSE, M. D., CINCINNATI, OHIO.

---

Who called the attention of the profession to the importance of making a more careful examination of every case where there is bleeding from the rectum. He stated that rectal hemorrhage must not be considered conclusive of the existence of piles. Many other diseases besides piles are accompanied with bleeding. He laid great stress on the importance of diagnosing malignancy in its early stage so as to give the patient a better chance of recovery. Many cases of malignant disease of the rectum, whose only symptom is hemorrhage have been overlooked and the patient sacrificed, which would not have occurred had the family physician insisted upon a local examination, thereby diagnosing the disease in its incipiency before it had gone beyond the operable stage. He further stated that every patient is entitled to a thorough examination; and physicians are in duty bound to use all the means at their command to accomplish it. As Murray very aptly expressed himself: "Thus a case that today would be operable and a cure result, if diagnosed, would be inoperable in six months or a year and death result." The author reported numerous cases where a correct diagnosis had not been made on account of the negligence of the family physician. Some had been operated upon for bleeding piles which subsequently turned out to be cancer. He concluded his article with the statement, "that earlier recognition of malignancy would add materially to the future welfare of the patient which can be obtained by surgical measures, and it therefore behooves the general practitioner to be on his guard and examine carefully every case of bleeding so as to detect malignancy in its incipient stage."

### Ano-Rectal Affections of Infancy and Childhood.

BY A. J. ZOBEL, M. D., SAN FRANCISCO, CAL.

This paper briefly describes those ano-rectal affections of infancy and childhood which may appear on one's daily work or in consultation practice.

From the first hour after birth the ano-rectal region is of vast importance. At that time malformations may be determined and proper relief promptly afforded.

The various malformations were enumerated and briefly described. Some of these abnormalities pass unnoticed throughout a long life but others are the source of great discomfort and distress.

Mention was made that while hemorrhoids are common in adults the possibility of their presence in the young is rarely considered. Yet they may appear in children of tender years. The various causes for hemorrhoids in the young were reviewed in this paper.

Malignant growths of the rectum while rare are occasionally met with. Cases were quoted where the disease was found in children as young as five years of age.

Benign growths are more common. Adenoma is the most frequent of these. They are often diagnosed as internal hemorrhoids, and like them, may become strangulated. They may exist for some time and attain quite a size without producing any symptoms until strangulation occurs.

Fissure of the anus is believed by the writer to be present more often than it is usually diagnosed. It may cause severe crying in nurslings. May cause reflex symptoms to appear which for a time may baffle the diagnostician. Some of these may resemble coxalgia. The incautious and improper introduction of syringe nozzles and thermometers into the anal canal frequently cause fissures. Other causes were also mentioned.

Especial stress was laid on the subject of pruritus ani in children. The writer believing it to be a very frequent source of great discomfort and torment to the little ones. It is very rarely suspected or diagnosed, and he believes that it accounts for much of that peevishness in these little ones for which no cause can usually be assigned. The child is seen to rub his anal region, saying "It hurts." Does not complain of itching. Seems to misinterpret the sensation. He has found superficial lesions of the anal mucous membrane in these cases, and as the symptoms disappeared when local treatment was instituted he feels assured that these were the cause of the trouble.

Fistulo-in-ano is met with occasionally in children and even in nurslings. While it may be tubercular, it may also be of a congenital nature.

Ischio-rectal abscesses are met with even in early infancy. When incised they rarely end in fistulae.

Prolapse of the mucous membrane of the anus and rectum is a common condition during the second and third years of life. Long continued tight binding in babyhood may be the starting point. Diarrhea is the most common antecedent. Anything that induces prolonged and severe straining at stool may be a cause. Some of these causes were mentioned.

The varieties and causes of proctitis were also dwelt upon. Proctitis is often taken for ordinary catarrhal diarrhea due to improper feeding. It is advised that when a gonorrhea of the genital tract exists in children that a secondary infection of the ano-rectal region should always be considered.

It is hoped that this reminder that infants and children have ano-rectal troubles, as well as adults, will lead to more thought being given in this direction, and that it will bear fruit in bringing relief to some of these little sufferers.

### The Treatment of Rectal Fistula.

BY J. RAWSON PENNINGTON, M. D., CHICAGO, ILL.

Who referred to three methods, viz.: simple incision; the injection of bismuth paste; the incision or excision with immediate suture (Proctorrhaphy).

Of the *simple incision* he said: Those of us who are operating quite frequently for this malady know its disadvantages, drawbacks, and frequent failures to cure. That this operation has done more than any other, unless it be that of the ligature or clamp and cautery operation for hemorrhoids, to bring disrepute upon rectal surgery. That the laity dread a rectal operation more than any other surgical procedure because of the fear of pain, the fear of recovery and the fear of loss of control over the bowels. Yet, we know that each of the above operations in the hands of experts give good results. Concerning the injection of bismuth paste, he said: To treat a rectal fistula, the paste is liquefied by heating in a water-bath and injected into one of the opening with a metal or glass syringe. The other opening or openings are kept closed by an assistant while the injection is being made. Enough force is used until one feels reasonably sure that all tracts and diverticuli have been filled. The paste may be forced into some line of cleavage if too much tension is used and carried along this line to some distant organ or healthy tissue and deposited there with deleterious results.

Of excision or incision with immediate suture (Proctorrhaphy), he said: This method is the most rational of all surgical procedures; that he dissects and removes the entire tract when a probe or director can be passed through the fistulous channel and into the rectum. That he then searches out and removes any diverticuli or tracts connected with the main tract. If this can not be, or should not be done, he then incises the fistula and dissects out all granulation tissue. If needs be the wound is disinfected with carbolic acid and alcohol.

Suturing the wound may be done by lembertizing the line of incision from its termination in the rectum to the anus. The ends of the several sphincters as well as the deeper portions of the incision are next brought together with interrupted catgut sutures. The skin and fascia are sutured with interrupted silkworm gut. He dresses the wound with iodoform or plain gauze and applies a T bandange. He maintains that Proctorrhaphy, or the paste, or a combination of the two, offers the nearest approach we have to the ideal method of treating extensive rectal fistulae.

---

### The Tuberculin Reaction in Cases of Peri-rectal Infection.

---

BY COLLIER F. MARTIN, M. D., PHILADELPHIA, PA.

---

The author was so impressed with the frequent coincidence of pulmonary tuberculosis and peri-rectal infections that he began a series of tests and examinations to determine their relation.

He uses the Moro tuberculin reaction, combined with physical and bacteriologic examination.

In his preliminary report of thirty-six cases, which he divides into two groups, he got the following results:

*Group I.*—Rectal pyogenic infections, including here fistulae, abscesses, and deep rectal ulcerations. There were twenty positive reactions out of twenty-one cases. The negative case was one profoundly tuberculous.

*Group II.*—Non-pyogenic rectal cases. There were eleven cases, including hemorrhoids, fissure, and catarrhal proctitis, with three positive tuberculin reactions. This, he holds, is probably the ratio of tuberculosis in this class of cases. One negative case in this group was intensely tubercular, with extensive lung lesions evident, and with abundant tubercle bacilli in the sputum.

Accepting the tuberculin test as a specific one, he got 100 per cent positive in Group I, and about 36 per cent in Group II. The four cases giving negative reactions, yet being proved tuberculous, by sputum examination, proved to be of very low resistance, two dying in a few months and two, at present, in a percarious condition.

He emphasizes "continued history taking" as being extremely valuable to the proper appreciation of the case.

The author places particular stress on the prognostic value of the tuberculin test.

Accepting the positive reaction to tuberculin as indicative of a tuberculous lesion somewhere in the body, his conclusions are as follows:

1. Two consecutive, negative reactions, with no physical signs in evidence, is conclusive proof of the absence of such lesions.

2. Two consecutive negative or feeble reactions, with physical signs of a lesion somewhere, is indicative of a very grave prognosis.

3. The degree of the reaction is directly proportionate to the degree of the resistance of that individual.

4. That the tubercle bacillus, like no other, reduces the bodily defenses to pyogenic invasion.

5. That in practically all rectal pyogenic infections there is a tuberculous lesion somewhere in the body.

6. That the classification of peri-rectal infections into tuberculous and non-tuberculous is untenable.

His investigations have caused the author to raise the following questions:

1. Is the primary tuberculous lesion pulmonary?

2. Is the local infection tuberculous?

3. Do the tubercle bacilli gain entrance into the body through the respiratory or the alimentary tract?

4. Is such infection carried to the rectal and peri-rectal tissues by the blood current, the lymphatics, or directly, by the fecal current?

5. How does the tubercle bacillus influence the pyogenic infections—locally, as in mixed infection, or by lowering the body-resistance to the invasion by pyogenic bacteria?

---

### Lane's Conception of Chronic Constipation and It's Management.

---

BY A. B. COOKE, M. D., NASHVILLE, TENN.

---

In his monograph entitled "The Operative Treatment of Chronic Constipation," Mr. Lane first defines the scope of the treatise by stating that the term, chronic constipation, as he employs it includes all those conditions which are "the consequences of the accumulation of material in the intestinal tract for a period sufficiently in excess of the normal to produce on the one hand alteration in the gastro-intestinal tract and in other viscera, and on the other hand toxic changes from absorption." The fact is emphasized that while constipation is usually marked by infrequent hard stools, there may be a daily evacuation, and in exceptional cases the motions are loose and frequent.

The two chief pathologic factors in the production of chronic constipation, according to the author, are enteroptosis and acquired mesenteries or adhesions, the latter resulting not from inflammation, but being developed to oppose the displacement of viscera, the tendency to which exists whenever the erect posture of the trunk is assumed. The displacement and fixation of the several portions of the colon in faulty positions result primarily in defective drainage, and secondarily in auto-intoxication and pathologic changes both in the gut itself and in the other abdominal viscera.

After describing these changes in detail, the author proceeds to discuss their immediate and remote effects, advancing the idea that in many cases diseases of the appendix, gall-bladder, stomach, duodenum, pancreas, kidneys, ovaries, etc., must be regarded as sequelae of chronic constipation. In addition to the phenomena resulting from toxic absorption are graphically described and the importance of their recognition stressed.

With reference to treatment Lane states that "in no circumstances should operative interference be contemplated till the surgeon has satisfied himself that every means of treatment has failed, whether medical or mechanical." The surgery indicated depends upon the conditions present. In mild cases in which non-operative measures have failed, division of the adhesions and constricting bands may be effective. Severer cases call for more radical surgery consisting either in dividing the ileum and anastomosing it with the sigmoid or upper rectum, thus short-circuiting the fecal current, or, when pain is a prominent factor in the case, removal of the colon in addition.

The writer of the paper, after personal observation of Lane's work, regards his conception of the nature and management of the malady with much favor and thinks it entitled to serious consideration at the hands of the profession.

---

### A Unique Case of Laceration of the Sphincter Ani.

BY A. B. COOKE, M. D., NASHVILLE, TENN.

---

On February 26, 1910, the patient, a boy seven years old, was brought to him at St. Thomas Hospital, accompanied by his father and physician. The following remarkable history was related: About noon on the day named the boy, who lived on a farm, went out to his favorite place behind the corn-crib to attend to a call of nature. While engaged in the act, a pet dog, a hound of middle size, came up from the rear and mounting him effected entrance to the anum and became accoupled. The boy's outcries quickly brought his mother upon the scene. The dog had reversed his position and was in the same relation to the boy as is ordinarily assumed in the natural act with a bitch. The mother's excitement was naturally marked and in her frantic efforts to disentangle the two she used considerable violence and finally succeeded in separating the dog.

The family physician on his arrival found that the hemorrhage had practically ceased, but upon inspection of the bowel found the parts badly lacerated and advised the patient's removal to Nashville for treatment.

Dr. Cooke's examination found very little evidence of external injury. Traction upon the anus, however, showed that several internal lacerations of considerable extent were present. Under general anesthesia the deepest of these was found to be in the middle line posteriorly, extending from a point two inches up the rectum through the sphincter muscles, and out upon the skin surface for a distance of approximately one inch. The external sphincter was torn in two places at this site, one tear being complete, and other partial. Anteriorly there was a second laceration, into but not through the fibers of the sphincter. In addition there was a number of minor tears in the anal margin involving the superficial tissue only.

Fourteen interrupted catgut sutures were used in repairing the posterior laceration, and four in the anterior one. The others did not require suturing. The result was entirely satisfactory. Union was prompt and complete and the patient returned home in two weeks with perfect sphincter control.

---

### An Open Letter Contributed to the New York Times By Professor Frederic S. Lee.

There has been held in this city during the past ten weeks a public exhibition purporting to demonstrate the methods that are employed in laboratories of animal experimentation. It is held under the auspices of the New York Anti-Vivisection Society, an organization which for the past two years has endeavored in various ways to keep itself in the public eye. The exhibition has attracted less attention from the public than it deserves, for, while its scientific character may be questioned, it is valuable as affording a clue to the moral character of an organization which lays claim to a position of moral leadership. There are some of us who have entertained grave doubts as to whether this claim is justified, and these doubts have increased as the various successive acts of the society have been performed since the day of its birth. A study of its exhibition tends to increase these doubts.

The most graphic feature of the exhibition is an array of stuffed animals, some attached to operating tables, some with heads attached to surgical head-holders, some in partial dissection, with surgical instruments lying about, and in one case with a pool of red liquid, simulating blood. The good taste manifested in the public showing of such gruesome sights may well be questioned, and especially in view of the fact that a considerable number of the visitors which one sees at the exhibition are children. They are not only welcomed and allowed to roam freely about the room, but the unpleasant details of the exhibit are explained to them by the women attendants in charge, and a morbid curiosity is thus encouraged. The walls bear many pictures of animals, some undergoing luridly red surgical operations, some exhibiting anatomical dissections, and others participating in a variety of scenes of happiness and misery. The investigator is now and then shown, with a face of diabolical glee, gloating over his victim. A considerable number of portraits of men are shown, chiefly literary men and clergymen, with extracts from their writings, expressing more or less opposition to animal experimentation. In many cases these expressions are direct responses to requests by members of the society, and their language shows the degrading influence of the literature circulated by the society.

A significant part of the exhibition consists of the tales that are told to the visitors by the women attendants. Of the various operations that are portrayed or suggested, one is frequently told that they are customarily performed without anesthetics, a statement which is not true. One attendant said to a visitor that the surgical head-holders were used for the purpose of breaking the jaws of dogs, and that this was done without anesthetics. When questioned as to the reason for breaking the jaws of dogs she confessed ignorance. Such a procedure is so patently fantastic as to render comment unnecessary. There is an oven, heated by gas burners which contains the stuffed body of a rabbit, and which the attendant tells you is used for the purpose of baking live animals to death, and that this is also performed without anesthetics. Fabrication and grotesqueness here reach their culmination, for the oven is an apparatus intended for the incineration of dead organic matter, the anatomical refuse of a laboratory! The attendants are ever ready to discuss animal experimentation, seemingly quite unaware of their great and prejudiced ignorance. They do not hesitate to speak of well-known and highly respected scientific men in intemperate language that is anything but refined or parliamentary.

To one who is familiar with laboratory procedure, the keynote of this exhibition is falsity. The visiting layman can hardly fail to carry away with him a wholly incorrect notion of what animal experimentation means, what its methods are, and what a measureless amount of good it has accomplished for both the human race and the lower animals. Nowhere is there a sincere desire for the truth; everywhere there is ignorance, misrepresentation, and false implication; everywhere the calmness of balanced judgment is wanting; everywhere there is an unbridled appeal to sentiment, and to sentiment inflamed into passion. The harm is great that may thus be done to the individual, but when such an influence is allowed to spread unchecked through a community the harm that may be done to the multitude is incalculable. Such an influence is both intellectually and morally debasing. When a bishop of a Christian Church, innocent of the truth and moved only by a blind rage excited by the misleading tales of this society, writes of the beneficent method of animal experimentation, a method from which he and his followers unwittingly derive daily blessings. "I have long been an enemy to vivisection, and am so still, * * * I would like to see it totally abolished and made an offense against the law. * * * I am heartily in sympathy with the effort, not only to reform, but to destroy and root out altogether this sin against the lives of innocent creatures," we may well ask whether the time has not come for enlightened people to band themselves together in opposition to this variety of fatuous fanaticism.

In the exhibition of which I write the most striking single exhibit is the New York Anti-Vivisection Society itself. It has had every opportunity to learn the truth or the falsity of its demonstrations and its declarations. It has been told by those who know how untrue they are, and yet it has continued week after week to keep its deceptive sights before the public and to tell its false tales. In the minds of those who both know and respect the truth the New York Anti-Vivisection Society stands, under the deceitful mask of a pretended moral leader, as an obscurantist, a partisan of vicious principles and practices, and a foe of the public good.

FREDERIC S. LEE.

Columbia University, February 4, 1910.

---

### A Remarkable Advertisement.

Tombstone, Arizona, claims to have the frankest saloonkeeper in the United States. He keeps the Temple Bar Saloon and advertises his business in a remarkable manner. He has had cards printed bearing the following words:

"Friends and Neighbors: I am grateful for past favors and having supplied my store with a fine line of choice liquors, allow me to inform you that I shall continue to make drunkards, paupers and beggars for the sober, industrious, respectable part of the community to support. My liquors will excite riot, robbery and bloodshed.

"They will diminish your comforts, increase your expenses and shorten life. I shall confidently recommend them as sure to multiply fatal accidents and incurable diseases.

"They will deprive some of life, others of reason, many of character and all of peace. They will make fathers fiends, wives widows, children orphans and all poor. I will train your sons in infidelity, dissipation, ignorance, lewdness and every other vice. I will corrupt the ministers of religion, obstruct the gospel, defile the church and cause as much temporal and eternal death as I

can. I will thus 'accommodate the public'—it may be at the loss of my never-dying soul. But I have a family to support, the business pays and the public encourages it.

"I have paid my license and the traffic is lawful, and if I don't sell it somebody will. I know the Bible says: 'Thou shalt not kill, no drunkard shall enter the kingdom of heaven,' and I do not expect the drunkard maker to fare any better, but I want an easy living and I have resolved to gather the wages of iniquity and fatten on the ruin of my species.

"I shall, therefore, carry on my business with energy and do my best to diminish the wealth of the nation and endanger the safety of the State. As my business flourishes in proportion to your sensibility and ignorance, I will do my best to prevent moral purity and intellectual growth.

"Should you doubt my ability, I refer you to the pawnshops, the poorhouse, the police court, the hospital, the penitentiary and the gallows, where you will find many of my best customers have gone. A sight of them will convince you that I do what I say.

"Allow me to inform you that you are fools, and that I am an honest saloonkeeper."—*Lay Exchange.*

---

### The Charles H. Phillips Chemical Company.

As in previous years, this firm confined its exhibit to two of its products, the one being a compound syrup of quinine, the other a fluid magnesia. The former is known as the Syrup of Phospho-Muriate of Quinine Compound, its strong point being that in its preparation the muriate instead of the sulphate of quinine is used, and phosphates instead of hypophosphites. Hence, the preparation being acid, there is no risk of the contained strychnine being thrown down, as sometimes occurs in the case of hyphophosphite syrups. It is a pleasant bitter tonic, not productive of headache, and very stable. The fluid magnesia of the firm is termed Milk of Magnesia, this being the registered title of an odorless, white, palatable fluid with the physical appearance of milk. It is a hydrated oxide of magnesia, each fluid ounce representing, we understand, magnesium hydrate 24 grains. Under the microscope it is seen to be homogeneous, a fact which supports the firm's statement that their Milk of Megnesia is not, as are many magnesia preparations, merely a triturated magnesia suspended by mucilaginous or glycerine solutions. It attributes its special value as a neutralizer of free acids to the fact that it is entirely free from carbonates, and therefore does not give rise to discomforting evolutions of carbonic acid gas. It combines readily with tinctures as well as with iodides and other solutions of salts, and is useful as a suspender of fixed and volatile oils. We have had considerable experience of its use in the diarrhea of children and in gastric irritability, and consider it an excellent form in which to administer magnesia when indicated in such cases. It may be also substituted for lime-water in the modification of cow's milk. Owing to its presistent alkilinity and tastelessness it forms a good mouth-wash for use at bedtime.—*British Medical Journal, London.*

---

### He Had a Brother at Harvard, All Right.

Timothy Olcott, an urchin of wretched appearance, was haled before a Boston magistrate, charged with obstructing traffic by playing ball in Tremont Street.

"Can't your parents dress you better than this?" the magistrate asked, looking with disgust at Timothy Olcott's filthy rags.

"Me parents is dead," Timothy blubbered.

"But you've got some friends, surely?" said the magistrate.

"I've got a brother," the boy answered. His brow cleared, and he spoke proudly.

"Where is he?"

"He's at Harvard University," said Timothy, throwing out his chest.

"Is he in a good position there?" asked the magistrate.

"No," said Tom. "He's in a bottle there. He was born with two heads."—*Exchange.*

